# Crystal structure of CyanoQ from the thermophilic cyanobacterium *Thermosynechococcus elongatus* and detection in isolated photosystem II complexes

**DOI:** 10.1007/s11120-014-0010-z

**Published:** 2014-05-18

**Authors:** Franck Michoux, Marko Boehm, Wojciech Bialek, Kenji Takasaka, Karim Maghlaoui, James Barber, James W. Murray, Peter J. Nixon

**Affiliations:** 1 Department of Life Sciences, Sir Ernst Chain Building-Wolfson Laboratories Imperial College London, South Kensington Campus, London, SW7 2AZ UK; 2 Present Address: Alkion Biopharma, 4 rue Pierre Fontaine, 91000 Evry, France

**Keywords:** Photosynthetic oxygen evolution, PsbQ, PsbQ’, CyanoP, Psb27, Peroxiredoxin

## Abstract

The PsbQ-like protein, termed CyanoQ, found in the cyanobacterium *Synechocystis* sp. PCC 6803 is thought to bind to the lumenal surface of photosystem II (PSII), helping to shield the Mn_4_CaO_5_ oxygen-evolving cluster. CyanoQ is, however, absent from the crystal structures of PSII isolated from thermophilic cyanobacteria raising the possibility that the association of CyanoQ with PSII might not be a conserved feature. Here, we show that CyanoQ (encoded by *tll2057*) is indeed expressed in the thermophilic cyanobacterium *Thermosynechococcus elongatus* and provide evidence in support of its assignment as a lipoprotein. Using an immunochemical approach, we show that CyanoQ co-purifies with PSII and is actually present in highly pure PSII samples used to generate PSII crystals. The absence of CyanoQ in the final crystal structure is possibly due to detachment of CyanoQ during crystallisation or its presence in sub-stoichiometric amounts. In contrast, the PsbP homologue, CyanoP, is severely depleted in isolated PSII complexes. We have also determined the crystal structure of CyanoQ from *T. elongatus* to a resolution of 1.6 Å. It lacks bound metal ions and contains a four-helix up-down bundle similar to the ones found in *Synechocystis* CyanoQ and spinach PsbQ. However, the N-terminal region and extensive lysine patch that are thought to be important for binding of PsbQ to PSII are not conserved in *T. elongatus* CyanoQ.

**Electronic supplementary material:**

The online version of this article (doi:10.1007/s11120-014-0010-z) contains supplementary material, which is available to authorized users.

## Introduction

The water oxidation reaction of oxygenic photosynthesis is catalysed by the photosystem II (PSII) complex located in the thylakoid membranes of chloroplasts and cyanobacteria. Crystal structures of monomeric and dimeric oxygen-evolving PSII complexes isolated from the thermophilic cyanobacteria *Thermosynechococcus vulcanus* and *Thermosynechococcus elongatus* have been determined (Kamiya and Shen 2003; Ferreira et al. 2004; Loll et al. 2005; Guskov et al. 2009; Broser et al. 2010; Umena et al. 2011). Each PSII monomer contains about 20 subunits, depending on the preparation, most of which are integral to the membrane (reviewed by Müh et al. 2008). In the case of cyanobacteria three extrinsic proteins (PsbO, PsbU and PsbV) are attached to the lumenal surface of the crystallised complex where in vivo they help to shield the Mn_4_CaO_5_ oxygen-evolving complex from aberrant reduction (Shen et al. 1998). A different set of proteins (PsbO, PsbP, PsbQ and PsbR) is associated with PSII in green algae and higher plant chloroplasts, but their binding sites remain unclear (reviewed by Bricker et al. 2012). For red algae and diatoms, an intermediate situation exists in which a PsbQ-like subunit (termed PsbQ’) is present in addition to the PsbO, PsbU and PsbV subunits, while a fifth subunit, Psb31, is also found in diatoms (reviewed by Enami et al. 2008). PsbP-like and PsbQ-like proteins are also expressed in higher plant chloroplasts, but they have roles outside PSII. For instance, two PsbQ-like proteins are components of the thylakoid NADH dehydrogenase-like (NDH) complex in *Arabidopsis* (Yabuta et al. 2010).

Homologues of PsbP and PsbQ are also found in cyanobacteria (Thornton et al. 2004). The function of these two proteins, designated here as CyanoP and CyanoQ, respectively, is still obscure (reviewed by Fagerlund and Eaton-Rye 2011), particularly as they are not present in the published crystal structures of PSII (Kamiya and Shen 2003; Ferreira et al. 2004; Loll et al. 2005; Guskov et al. 2009; Umena et al. 2011). Most work on the structure and function of CyanoQ has come from studies of the mesophilic cyanobacterium *Synechocystis* sp. PCC 6803, hereafter *Synechocystis*, where it is known to be a subunit of oxygen-evolving PSII complexes (Roose et al. 2007). *Synechocystis* cells lacking CyanoQ grow photoautotrophically as well as WT under optimal growth conditions but do show some growth inhibition when exposed to nutrient stress such as by depleting the medium of calcium and chloride (Thornton et al. 2004) and iron (Summerfield et al. 2005). Analysis of isolated PSII complexes lacking CyanoQ from *Synechocystis* suggests that CyanoQ stabilises binding of PsbV and helps protect the oxygen-evolving Mn_4_CaO_5_ complex from reduction in the dark (Kashino et al. 2006).

The crystal structure of *Escherichia coli*-expressed *Synechocystis* CyanoQ, determined to a resolution of 1.8 Å, is similar to that of PsbQ from spinach with a root mean square deviation (RMSD) for the C_α_ atoms of 1.4 Å despite only 17 % identity in primary structure (Jackson et al. 2010). Both crystallised proteins consist of a four-helix bundle and contain bound Zn^2+^, although a metal-free structure has also been determined for *Synechocystis* CyanoQ (Jackson et al. 2010); the physiological relevance of these metal-binding sites is currently unknown. In contrast, much less is known about CyanoQ in the thermophilic cyanobacteria used for structural studies of PSII. Indeed the association of CyanoQ with PSII in either *T. elongatus* or *T. vulcanus* has yet to be demonstrated. Here, we describe the crystal structure of *E. coli*-expressed CyanoQ from *T. elongatus* and provide evidence that CyanoQ co-purifies with isolated PSII and strikingly is still present in samples used to generate PSII crystals lacking CyanoQ.

## Materials and methods

### *Thermosynechococcus elongatus* BP1 strains

A His-tagged CP43 strain (CP43-His) of *Thermosynechococcus elongatus* (Sugiura and Inoue 1999) was kindly provided by Dr Miwa Sugiura, and a His_6_-tagged derivative of CP47 (CP47-His) by Dr Diana Kirilovsky. The WT strain was the same as that used by Ferreira et al. (2004).

### Construction of plasmid for over-expression of CyanoQ

The DNA sequence corresponding to the CyanoQ homologue of *T. elongatus* (tll2057) without the sequence encoding the predicted signal peptide and lipid-binding Cys24 residue was cloned into a pRSET-A vector modified as described in Bialek et al. (2013). The corresponding PCR fragment was amplified from *T. elongatus* genomic DNA using Phusion polymerase (NEB, UK) and primers CyanoQ-XhoI-F (5′-TATATACTCGAGGGCGGCCCCAGTGCCACCACTCCACCCCCACCCACCTA-3′) and CyanoQ-EcoRI-R (5′-TATATAGAATTCTTACTAGGACAACTCAGGCAAGCTGTTGAGAT-3′) introducing underlined restriction sites, double digested with XhoI and EcoRI and ligated (Quick Ligation Kit, NEB, UK) into the modified and XhoI/EcoRI linearised pRSET-A. The vector was then transformed into KRX *E. coli* cells (Promega, UK).

### Expression, purification and crystallisation of CyanoQ

Expression of His_6_-tagged CyanoQ was induced by the addition of 2 g/L of rhamnose, and cells were grown at 18 °C overnight. Cells were lysed with a sonicator (Sonics and Materials, CT, USA) in lysis buffer (50 mM Tris–HCl pH 7.9, 500 mM NaCl, 1 mM MgCl_2_) supplemented with one Complete Protease Inhibitor Cocktail-EDTA Tablet (Roche, UK) per 50 ml lysis buffer. Broken cells were spun down for 10 min at 4 °C at 18,000×*g*, and the supernatant was mixed with a Ni-iminodiacetic acid resin (Generon, UK). Non-specifically bound proteins were removed by washing 3 times with wash buffer (20 mM Tris–HCl pH 7.9, 500 mM NaCl, 60 mM imidazole), and His_6_-CyanoQ was eluted with elution buffer (20 mM Tris–HCl pH 7.9, 500 mM NaCl, 1 M imidazole). Purified His_6_-CyanoQ was dialysed overnight against 20 mM Tris–HCl pH 7.9, 200 mM NaCl at 4 °C. The His-tag was removed by thrombin (GE Healthcare, UK) digestion at a ratio of 1 unit of thrombin per 100 µg of purified CyanoQ. Proteolysis was performed overnight at 4 °C and the digested sample was reloaded onto a nickel-iminodiacetic acid column. The flow-through containing CyanoQ without the His-tag was concentrated at 4 °C to around 10 mg/ml with a centrifugal concentrator device with a molecular weight cut off (MWCO) of 3500 (Sartorius, Germany). Crystals appeared in hanging drop vapour diffusion, above 1.8 M ammonium sulphate, with drops of protein solution and an equal volume of mother liquor. Crystals were cryoprotected in the mother-liquor solution with 30 % (v/v) glycerol, then flash-cooled in liquid nitrogen.

### Protein structure determination

Data were integrated and scaled with MOSFLM (Leslie and Powell 2007) and programmes of the CCP4 suite (Winn et al. 2011). 5 % of reflections were set aside as the Free set for cross-validation. The structure was solved by molecular replacement using the CyanoQ structure from *Synechocystis* (Jackson et al. 2010). The model was truncated using Chainsaw (Stein 2008) mode, and used as a model in PHASER (McCoy et al. 2007). The structure was refined in REFMAC (Murshudov et al. 2011) with cycles of manual model-building in COOT (Emsley and Cowtan 2004). Validation was performed using the MolProbity server (Davis et al. 2007). The atomic model and structure factors have been deposited in the PDB under accession number 3ZSU.

### Sequence alignment and structural conservation

The full protein sequence of CyanoQ (Tll2057) from *T. elongatus* was searched against cyanobacterial genomes using BLAST (Altschul et al. 1990) having gapless chromosome assembly level on NCBI. Sequences were aligned in ClustalW2 and analysed by Prosite (De Castro et al. 2006).

## Isolation of PSII complexes from *T. elongatus*

*Thermosynechococcus elongatus* cultures were grown in DTN medium (Mühlenhoff and Chauvat 1996) at a constant temperature of 45 °C under continuous illumination (~60 μmol photons·m^−2^·s^−1^) by an Innova 44 shaker (New Brunswick Scientific) at 120 rpm. Typically 5-L Erlenmeyer flasks were used to grow five 3.5-l cultures to give a total culture volume of about 17.5 l. Cells were harvested at an optical density of about 1 at 750 nm using a Sartocon cross flow filtration system (Sartorius) followed by centrifugation at 10,000 rpm (JA14 rotor, Beckman Coulter Ltd.) for 5 min at room temperature. The cell pellet was re-suspended in RSB buffer (40 mM MES–NaOH pH 6.5, 15 mM MgCl_2,_ 15 mM CaCl_2_, 1.2 M betaine and 10 % (v/v) glycerol) to a volume of 50–75 ml and disrupted by 2 passes at 25,000 psi using a T5 cell disruptor set to 4 °C (Constant Systems Ltd). Unbroken cells were removed by centrifugation at 1,000×*g* (JA14 rotor, Beckman Coulter Ltd.) for 5 min at 4 °C, and membranes were pelleted and washed three times with the same buffer by centrifugation at 184,000×*g* (Ti45 rotor, Beckman Coulter Ltd.) for 20 min at 4 °C. Membranes were then resuspended in 20 mM MES–NaOH pH 6.5, 10 mM MgCl_2_, 20 mM CaCl_2_, 25 % (v/v) glycerol and stored at −0 °C. These membranes were then used to isolate PSII oxygen-evolving complexes from WT *T. elongatus* using the two-step anion-exchange chromatography procedure described by Kern et al. (2005).

Dimeric His-tagged oxygen-evolving complexes were isolated from a His-tagged CP47 strain of *T. elongatus* by Ni-affinity purification followed by anion-exchange chromatography as described by Nowaczyk et al. (2006) except for the following modifications: freshly grown cells were broken in 20 mM MES–NaOH pH 6.5, 2.5 mM CaCl_2_, 2.5 mM MgCl_2_, 10 % (v/v) glycerol and 1.2 M betaine, and unbroken cells were removed by centrifuging at 1,000 *g* (JA14 rotor, Beckman Coulter Ltd.) for 5 min at 4 °C; the resulting supernatant was diluted to a Chl concentration of 1 mg/ml and the thylakoid membranes were solubilised for 10 min at 4 °C with 1 % (w/v) n-dodecyl-β-D-maltoside (β-DDM) at a detergent to Chl ratio of 18:1 followed by a 30-min spin at 4 °C and 184,000 *g* (Ti70 rotor, Beckman Coulter Ltd.); the extract was incubated for 45 min with Ni-affinity resin (Probond Resin, Invitrogen) equilibrated in buffer E (20 mM MES–NaOH pH 6.5, 2.5 mM CaCl_2_, 2.5 mM MgCl_2_, 0.5 M D-mannitol and 0.03 % (w/v) β-DDM); after loading, the Ni-affinity column was washed with 6 column volumes of buffer E + 5-mM histidine; His-tagged PSII complexes were eluted by application of a 100-mM histidine isocratic step gradient in buffer E and loaded directly onto a Bio-Rad UNO Q-12 column using a AKTA Purifier 10 system (GE Healthcare Life Sciences); PSII complexes were eluted through the application of a 5–200-mM MgSO_4_ gradient in buffer E (at 2 mM/min and 4 ml/min). The third peak containing active PSII dimeric complexes (Nowaczyk et al. 2006) was concentrated using Vivaspin centrifugal concentrators (100,000 MWCO) before storing at −80 °C.

### Preparation of crude thylakoid membranes and pull-down experiments

Pull-down experiments were done using the His-tagged CP43 strain of *T. elongatus* and cobalt resin prepared by charging chelating Sepharose fast flow resin according to the manufacturer’s instructions (GE Healthcare Life Sciences). Crude thylakoid membranes were prepared from *T. elongatus* by glass bead breakage and differential centrifugation as described by Boehm et al. (2009) and re-suspended in buffer A (50 mM MES–NaOH pH 6.0, 10 mM MgCl_2_, 5 mM CaCl_2_, 10 % (w/v) glycerol) as used by Kashino et al. (2002). Thylakoids were solubilised with 1 % (w/v) β-DDM at a Chl concentration of 0.2 mg/ml for 10 min on ice in a final volume of 0.5 ml. After pelleting insoluble material by centrifuging in a microfuge, 0.45 ml of the supernatant was removed and diluted by addition of 0.45 ml of buffer A to which was added 0.1 ml of cobalt resin (50 µl of resin resuspended to final volume of 100 µl by addition of buffer A). Samples were then incubated on a rotating wheel at 4 °C for 2 h. After removal of the membrane extract, the cobalt resin was washed four times with 500 µl of buffer A, with the final wash kept for analysis. Bound proteins were eluted with 100 µl of buffer A containing 100-mM imidazole followed by 100 µl of 1× SDS sample buffer used for electrophoresis. Chelating Sepharose lacking bound metal ions was used as a control.

### Salt washes of purified PSII complexes and thylakoid membranes

PSII complexes in buffer A2 (20 mM MES–NaOH pH 6.5, 1 mM MgCl_2_, 1 mM CaCl_2_, 10 % (w/v) glycerol, 0.03 % (w/v) β-DDM) purified either by two-step anion-exchange or by nickel-affinity chromatography were incubated with buffer A2 supplemented with 1 M CaCl_2_ on ice for 30 min in the dark. Immediately after incubation samples were concentrated on 100,000 MWCO Vivaspin 500 centrifugal concentrators (Sartorius AG). Green retentate and flow-through containing removed extrinsic proteins were desalted by two buffer exchanges using Vivaspin 500 centrifugal concentrators, with MWCO of 100,000 and 3,000, respectively. Chlorophyll concentration was adjusted to 1 mg/ml and the volume of the filtrate was adjusted to match the volume of the green retentate. In the case of thylakoid membranes, proteins were extracted by high salt or high pH using the Freeze–Thaw approach described by Boehm et al. (2009).

### Protein analysis, isolation of protein and immunoblotting

*Thermosynechococcus elongatus* CyanoP and Psb27 were over-expressed in *E. coli* and purified as described previously (Michoux et al. 2010, 2012). These proteins plus CyanoQ isolated here were used to raise antibodies in rabbit. Protein samples were separated on 18 % (w/v) polyacrylamide gels containing 6 M urea as described by Boehm et al. (2009). Immunoblotting analyses were performed as described by Boehm et al. (2009) using the following antibodies and dilutions: αD1 (1:5000), αPsbO (1:1000), αCyanoP (1:2500), αCyanoQ (1:5000) and αPsb27 (1:2500). Chlorophyll *a* (Chl *a*) content and protein concentrations (using BSA as standard) were determined as described by Boehm et al. (2009). For densitometry gels were analysed by Image Studio Lite (LI-COR, Inc).

## Results and discussion

### CyanoQ associates with PSII complexes isolated from *T. elongatus*

**Fig. 1:**
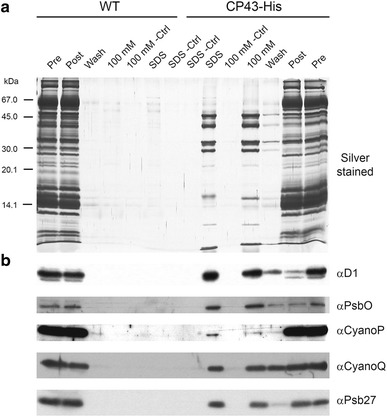
Association of CyanoQ with His-tagged CP43. Detergent solubilised membrane extracts from either WT or His-tagged CP43 strains of *T. elongatus* (CP43-His) were mixed with cobalt resin and the bound proteins eluted by 100-mM imidazole (100 mM) followed by SDS solubilising buffer (SDS) for analysis by **a** SDS-PAGE and silver staining and **b** immunoblotting. *Pre* solubilised extract added to resin; *Post* solubilised extract after incubation with cobalt resin; *Wash* last wash before elution; *Ctrl* control in which resin lacking Co was used

The CyanoP and CyanoQ orthologues in *T. elongatus* are encoded by *tlr2075* (Michoux et al. 2010) and *tll2057*, respectively. Despite detailed analysis of the subunit composition of His-tagged PSII complexes isolated from *T. elongatus* by mass spectrometry (Sugiura et al. 2010), neither CyanoQ nor CyanoP has been detected. To investigate whether CyanoQ or CyanoP are able to associate with PSII isolated from *T. elongatus*, we first performed pull-down experiments by binding solubilised membrane extracts obtained from a His-tagged CP43 strain of *T. elongatus* (CP43-His) to a cobalt resin and analysing bound proteins released by 100-mM imidazole. Immunoblotting experiments revealed that a significant proportion of CyanoQ co-purified with CP43-His (Fig. 1). By contrast, no detectable CyanoQ bound to the cobalt resin when a non-tagged WT sample was tested. As expected, the D1 and PsbO subunits of PSII co-purified with His-tagged CP43, as did significant amounts of Psb27, which is known to be a component of non-oxygen-evolving PSII complexes (Nowaczyk et al. 2006; Grasse et al. 2011). In contrast only trace amounts of CyanoP co-purified with CP47-His under the experimental conditions used.

**Fig. 2:**
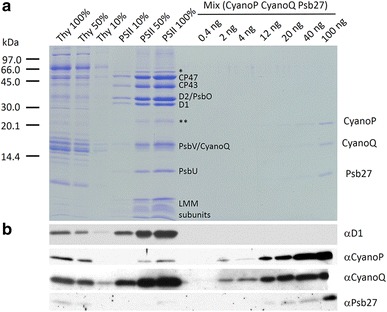
**a** SDS-PAGE analysis of serial dilutions of solubilised thylakoids membranes and *T. elongatus* PSII complexes isolated using the two-step anion-exchange chromatography method and known amounts of a mix of recombinant non-tagged CyanoP, CyanoQ and Psb27 proteins. 100 % level corresponds to 1 µg of Chl and amount in mix refers to amount of each of the proteins. Protein detected by Coomassie Blue staining. *Single asterisk* indicates migration of AtpA and *double asterisk* the migration of thioredoxin peroxidase as determined by mass spectrometry. Assignment of PSII subunits was determined through immunoblotting and mass spectrometry. *LMM* low molecular mass subunits of PSII. **b** Semi-quantitative immunoblotting analysis to determine CyanoP, CyanoQ and Psb27 levels in thylakoids and PSII

A commonly used method to isolate highly active oxygen-evolving dimeric PSII complexes from *T. elongatus* for structural studies involves a two-step anion-exchange chromatography protocol (Kern et al. 2005). This type of preparation has been successfully used to generate high-quality PSII crystals yielding diffraction data of up to 3 Å resolution (Loll et al. 2005; Murray et al. 2008a, b). The PSII preparation analysed here (which produced 400-µm-long PSII crystals) also contained detectable levels of the alpha subunit of the ATPase (Tlr0435) and, interestingly, a predicted thioredoxin peroxidase/peroxiredoxin (Tll1454), which is homologous to a peroxiredoxin (2-CysPrx) thought to interact with PSII in chloroplasts (Muthuramalingam et al. 2009) (Fig. 2). Immunoblotting of the PSII complex revealed that CyanoQ was indeed present and had been purified to about the same degree as the D1 subunit (approximate 10-fold enrichment on chlorophyll basis compared with thylakoid membranes). The presence of CyanoQ, which comigrates with PsbV upon SDS-PAGE, was confirmed by mass spectrometry (data not shown). In contrast less than 5 % of CyanoP and Psb27 originally found in the membrane were retained in the PSII fraction. More detailed analysis of individual fractions by immunoblotting confirmed that CyanoP and Psb27 had been removed during purification of dimeric PSII whereas CyanoQ co-purified (Figs. S1, S2). Loss of CyanoP on purification of PSII is in line with earlier studies on *Synechocystis* (Ishikawa et al. 2005).

We attempted to estimate the stoichiometry of CyanoQ present in the isolated PSII complex using a semi-quantitative immunoblotting approach (Fig. 2). A number of assumptions are made in this method including equal cross-reactivity of the native protein and *E. coli*-expressed version and the use of a protein assay to determine the amount of the standard; however this method has been applied previously to estimate levels of CyanoP and CyanoQ in *Synechocystis* (Thornton et al. 2004). Using the recombinant protein standards, we tentatively estimate that 20 ng of CyanoQ is present in PSII protein complexes containing 0.1 μg of Chl *a*. Assuming 35 Chl *a* are bound per PSII monomer and a molecular mass of 14,329 Da for CyanoQ, this would mean a CyanoQ:PSII monomer ratio of 0.4:1. In the case of *Synechocystis*, estimates range from 1.2 CyanoQ per 1 CP47 in membranes, determined by immunoblotting (Thornton et al. 2004), to approximately 0.25–0.30 CyanoQ per PSII based on the yield of His-tagged CyanoQ-containing PSII complexes (Roose et al. 2007). For CyanoP (molecular mass of 18,031 Da), assuming 1.3 ng of protein is present in PSII complexes containing 0.5 μg of Chl *a* (Fig. 2), the same calculation suggests that less than 1 % of PSII complexes in our preparation contain CyanoP.

Overall these data suggest that CyanoQ in *T. elongatus* co-purifies with dimeric PSII when isolated by anion-exchange chromatography. Absence of CyanoQ in PSII crystals obtained from this type of preparation could be due to detachment during crystallisation, such as by high salt (Fig. S3), or the fact that only PSII complexes lacking CyanoQ crystallised under the conditions tested. Importantly, we also found that the amount of CyanoQ associated with isolated PSII is variable with much less CyanoQ present in His-tagged PSII complexes isolated by the immobilised metal affinity chromatography and anion-exchange chromatography method described by Nowaczyk et al. 2006 (Fig. S3), which might explain why CyanoQ had not until now been detected in isolated His-tagged PSII complexes. In contrast, we have so far been unable to find conditions where CyanoP remains fully attached to PSII complexes.

### CyanoQ is a likely lipoprotein in *T. elongatus*

Like the situation in *Synechocystis* (Ujihara et al. 2008), both CyanoP and CyanoQ from *T. elongatus* contain a characteristic lipobox sequence, as detected by Prosite (De Castro et al. 2006), suggesting that they might be processed at the N-terminus and anchored to the membrane via lipidation of a cysteine residue (Fig. S4). Previous membrane washing experiments using either a high-salt treatment (2 M NaCl or 1 M CaCl_2_) or an alkaline treatment (pH 12.0), coupled with immunochemical detection, have shown that CyanoP is tightly bound to the membrane consistent with its assignment as a lipoprotein, whereas the non-lipidated extrinsic PsbO subunit is more easily removed (Michoux et al. 2010). Analysis of the same samples revealed that CyanoQ behaved like CyanoP and the lipidated Psb27 subunit of PSII (Nowaczyk et al. 2006) and was more resistant to extraction than PsbO (Fig. S5).

### Expression and crystallisation of the CyanoQ protein from *T. elongatus*

**Fig. 3:**
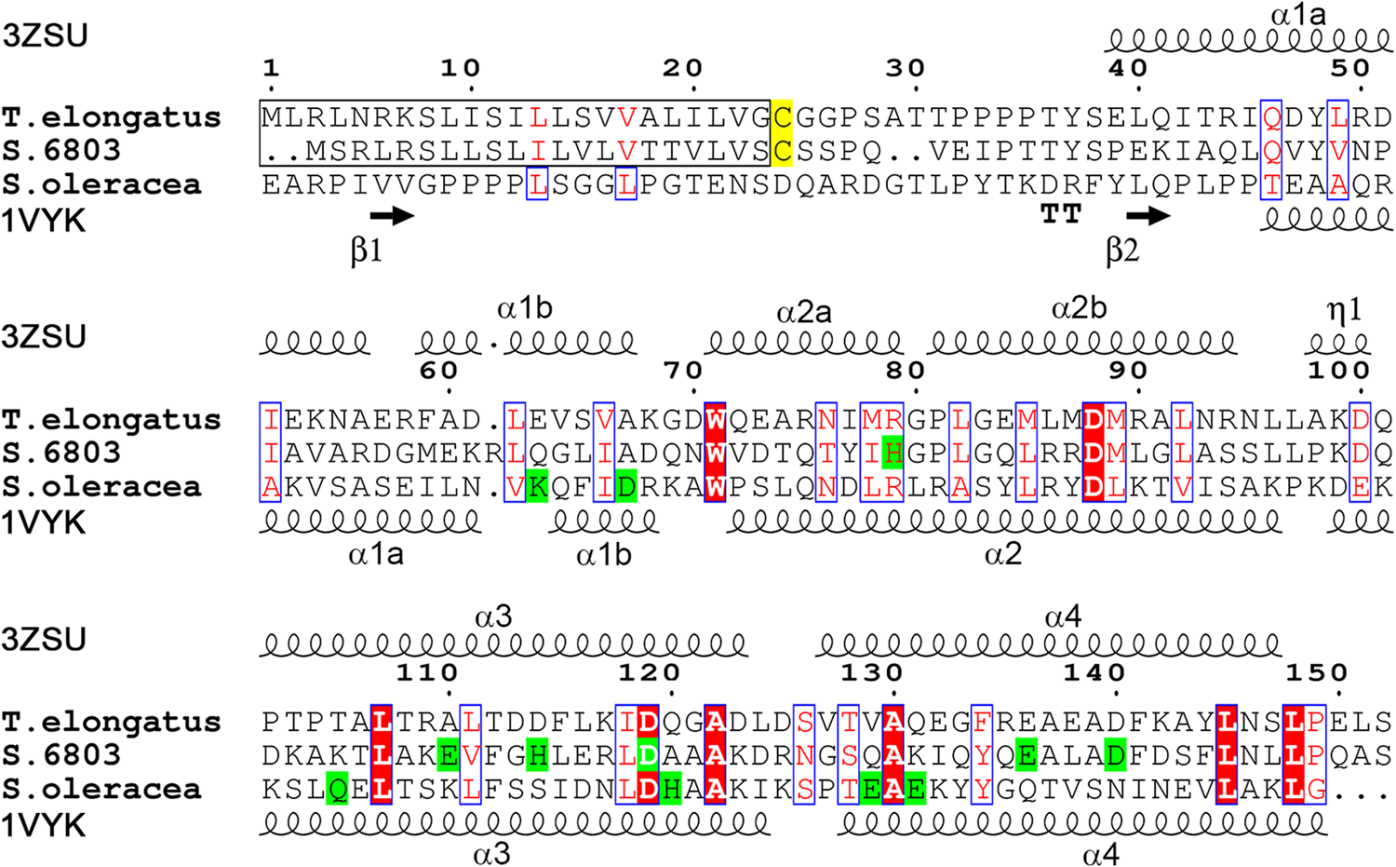
Sequence alignment of CyanoQ from *T. elongatus*, *Synechocystis* and PsbQ from spinach. Secondary structures are shown for CyanoQ from *T. elongatus* (3ZSU) and PsbQ from spinach (1VYK). Zinc-binding sites and lipidated cysteine residues are highlighted in *green* and *yellow*, respectively. Predicted signal peptides for CyanoQ are boxed in *black*. Numbering according to CyanoQ sequence from *T. elongatus*. Absolutely conserved and similar residues are shown as *white letters* on red background and *red letters* on white background, respectively, as calculated by ESPript (Gouet et al. 2003)

CyanoQ in *Synechocystis* and *T. elongatus* are relatively divergent with only 31 % sequence identity (Fig. 3 and Fig. S4). To gain insights into the structure of CyanoQ from *T. elongatus*, a cleavable N-terminal His_6_-tagged derivative lacking the predicted lipidated Cys_24_ (Fig. 3) residue was over-expressed in *E. coli* and the protein purified by immobilised nickel-affinity chromatography to near homogeneity (Fig. S6a). The His-tag was removed by thrombin cleavage and CyanoQ was re-purified and concentrated to 10 mg/ml (Fig. S6b). The predicted product contains residues 25–152 of CyanoQ plus 5 additional residues (GSELE) at the N-terminus. Crystallisation screens, performed using hanging drop plates, resulted in the formation of crystals, which were further optimised to grow in 1.8 M ammonium sulphate (Fig. S6c).

### Structural features and comparison with other CyanoQ proteins

**Fig. 4:**
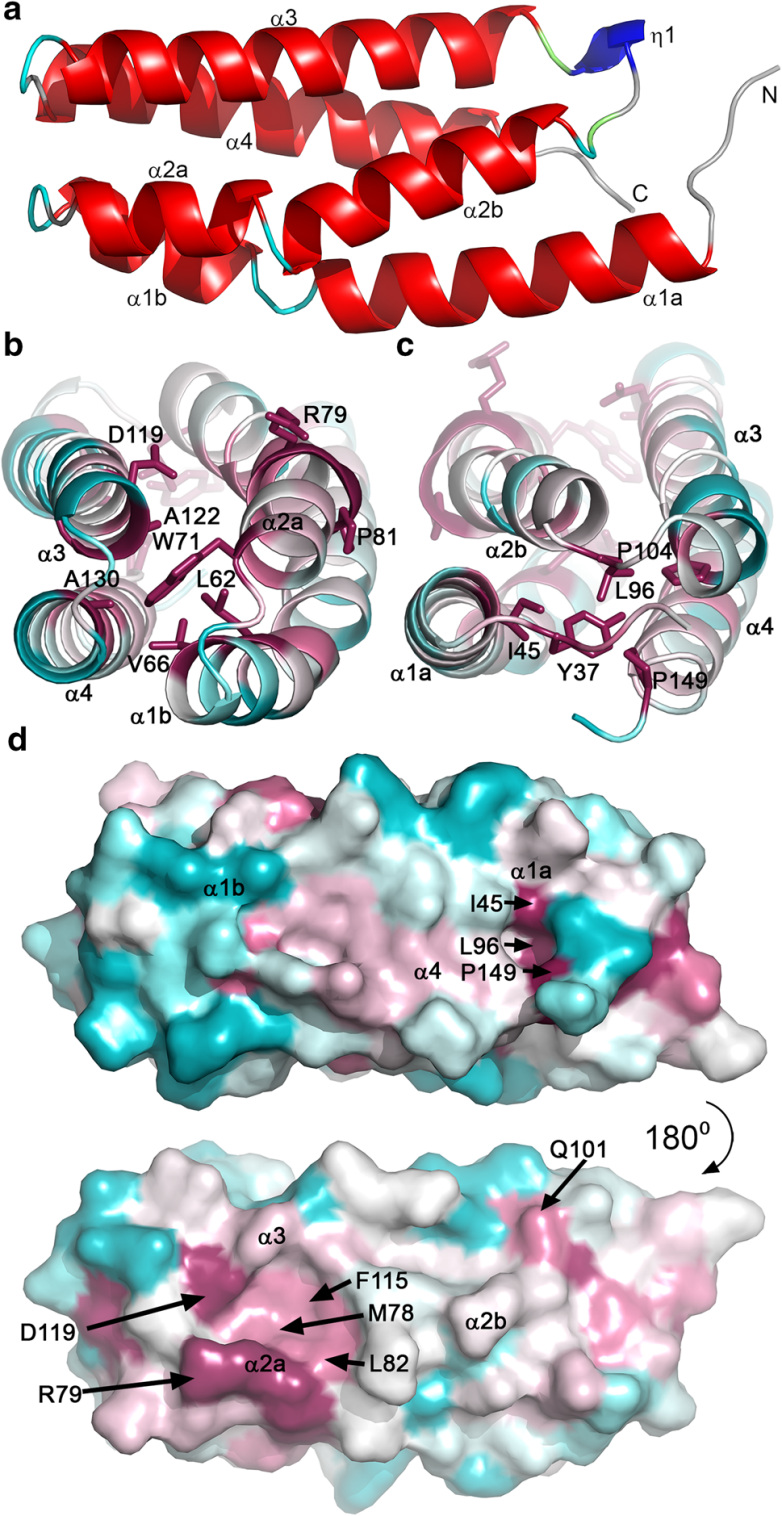
**a** Overall structure of CyanoQ from *T. elongatus* coloured according to DSSP (Kabsch and Sander 1983): α-helices (α1-α4, red), 3_10_ helix (blue, η1), hydrogen-bonded turns (cyan) and bends (green). **b** top and **c** bottom view of the protein coloured according to sequence conservation in cyanobacteria with most conserved residues shown as *sticks*. Bottom view in **c** corresponds to the end of CyanoQ containing the N- and C-termini. **d** Consurf (Ashkenazy et al. 2010) analysis of two conserved cavities (H4-H1 in upper view and H2–H3 in lower view; see text for details) with most conserved residues shown in *dark pink* and *magenta*. The most divergent regions are coloured in *cyan*

A native data set was obtained by X-ray crystallography at a resolution of 1.6 Å and the structure solved by molecular replacement using the crystal structure of CyanoQ from *Synechocystis* (PDB:3LS0, for details see Table 1). The refined co-ordinates of the 3D model of CyanoQ from *T. elongatus* have been deposited at the Protein Data Bank using the accession code 3ZSU. The first nine N-terminal residues as well as the last C-terminal residue of CyanoQ could not be detected in the electron density map so only residues 34–151 were fitted. Topologically the protein belongs to four-helix bundle superfamily and its fold is classified as mainly alpha up-down bundle (CATH 1.20.120.290) with four α-helices, of which the first two are broken, and one 3_10_ helix (Fig. 4a). The three-dimensional structure of CyanoQ from thermophilic *T. elongatus* showed a high level of similarity with the two structures of CyanoQ (with and without bound zinc) from the mesophilic *Synechocystis* (Jackson et al. 2010) with a RMSD of 1.6 Å for the *C*_α_ atoms (Table 2 and Fig. S7).

**Table 1:** Data collection and refinement statistics for the CyanoQ crystal structure

	CyanoQ data
X-ray source	Diamond I03
Data processing	Mosflm/Scala
Space group	P 21 21 21
Unit-cell parameters	*a* = 47.165 Å, *b* = 47.165 Å, c = 106.700 Å, *α* = *β* = 90°, *γ* = 120°
Wavelength (Å)	1.0722
Resolution (Å)	53.4–1.6 (1.69–1.60)
Measured reflections	130,767 (19,307)
Unique reflections	18,728 (2707)
Mn (I/sd)	10.8 (3.7)
Completeness (%)	99.38 (100.0)
Multiplicity	6.98 (7.13)
*R*_meas_ (%)	0.11 (0.62)
Solvent content (%)	48.6
*R*_work_/*R*_free_ (%)	16.7/19.0
Protein atoms	974
Solvent atoms	79
RMSD from ideal	
Bond lengths (Å)	0.022
Bond angles (°)	1.982
Average B factor (Å2)	18.2
Ramachandran favoured region (%)	100
Ramachandran allowed region (%)	0

$$R_{\text{meas}} = \mathop \sum \limits_{h} (\frac{n_{h}}{n_{h} - 1})\mathop \sum \limits_{I} I_{hl} - < I_{h} > /\mathop \sum \limits_{h} \mathop \sum \limits_{I} < I_{h} >$$

**Table 2:** Comparison of sequence identities and similarities (%, top) and structural RMSD (bottom) of CyanoQ from *T. elongatus* (3ZSU), *Synechocystis* with and without zinc (3LS1 and 3LS0) and PsbQ from spinach (1VYK and 1NZE)

	3ZSU	3LS0	3LS1	1VYK	1NZE
	*T. elongatus*	*Synechocystis*		*S. oleracea*	
3ZSU		31/50	31/50	14/24	14/24
3LS0	1.6 Å		100/100	17/33	17/33
3LS1	2.0 Å	0.7 Å		17/33	17/33
1VYK	1.6 Å	1.4 Å	1.6 Å		100/100
1NZE	1.5 Å	1.4 Å	1.6 Å	0.5 Å	

Although CyanoQ is likely to be lipidated in vivo in both *Synechocystis* and *T. elongatus*, this is not a universal feature of CyanoQ as the lipobox sequence and Cys residue needed for lipidation are absent in a number of other cyanobacteria (Fig. S4). These include *Acaryochloris marina*, a chlorophyll *d*-containing cyanobacterium and the siderophilic (having an affinity for iron) cyanobacterium JSC-12, whereas no protein homologous to CyanoQ could be detected in the *Prochlorococcus spp*., the two thermophilic species *Synechococcus sp*. JA-3-3Ab and *Synechococcus sp*. JA-2-3B’a(2-13) and the thylakoid-less *Gloeobacter violaceus* (De Las and Roman 2005; Fagerlund and Eaton-Rye 2011).

According to our sequence alignment, there are only two regions with absolutely conserved amino-acid residues across the cyanobacterial lineage. These regions flank helix 2a, the shortest one out of six found in this protein. The first amino-acid residue of helix 2a, Trp_71_, is absolutely conserved in the analysed CyanoQ sequences (Fig. S4). The indole nitrogen is exposed towards the solvent, and in this structure a 2.8 Å hydrogen bond is created between Trp_71_N^ε1^ and Asp_125_O^δ1^. A typical Ncap motif (Richardson and Richardson 1988) is observed for helix 2a where a main-chain carbonyl oxygen of Asp_70_ creates an hydrogen bond with the backbone amide nitrogen of Glu_73_. The other absolutely conserved residues are found right after the C-terminus of helix 2a and consist of a Gly_80_Pro_81_ motif that is immediately preceded by a positively charged amino acid, either arginine as in *T. elongatus* or in most cases histidine. Both glycine and proline are well known as the most efficient ‘helix breakers’ and in fact they separate helix 2a from helix 2b in CyanoQ (Fig. 4a).

Strongly conserved residues are found at both the apex and the base of the protein (Fig. 4b, c). Interestingly, these residues seem to shield the interior from the solvent by capping both ends of the protein. In agreement with the *Synechocystis* structures, we also observe two cavities, termed the H4-H1 and H2-H3 cavities by Jackson et al. (2010), composed of well-conserved residues (Fig. 4d). The smaller H4-H1 cavity is formed by Ile_45_, Leu_96_ and Pro_149_. In the case of *T. elongatus* the larger H2-H3 cavity is composed of a cluster of Met_78_, Arg_79_, Leu_82_, Phe_115_ and Asp_119_ surrounding the Gly_80_Pro_81_ motif. In the vicinity of this cavity, but absent in our structure, is found one of the Zn^2+^ ions in *Synechocystis* CyanoQ (Jackson et al. 2010).

## Comparison of CyanoQ and PsbQ

Currently there are two available structures of PsbQ from higher plants, both from spinach. The earlier structure (Calderone et al. 2003) lacks the first 37 residues whereas the later structure (Balsera et al. 2005) contains thirteen of these residues. Despite the low sequence similarity to spinach PsbQ, both CyanoQ and PsbQ are structurally similar (Table 2). One notable difference is the absence of the GlyPro helix breaking motif in algal or plant PsbQ sequences. As a consequence, the spinach structure shows a single 25 residue-long helix rather than the two helices (2a and 2b) observed in CyanoQ. In addition, PsbQ contains a much longer N-terminal sequence, which might be important for binding to PSII (Kuwabara et al. 1986).

**Fig. 5:**
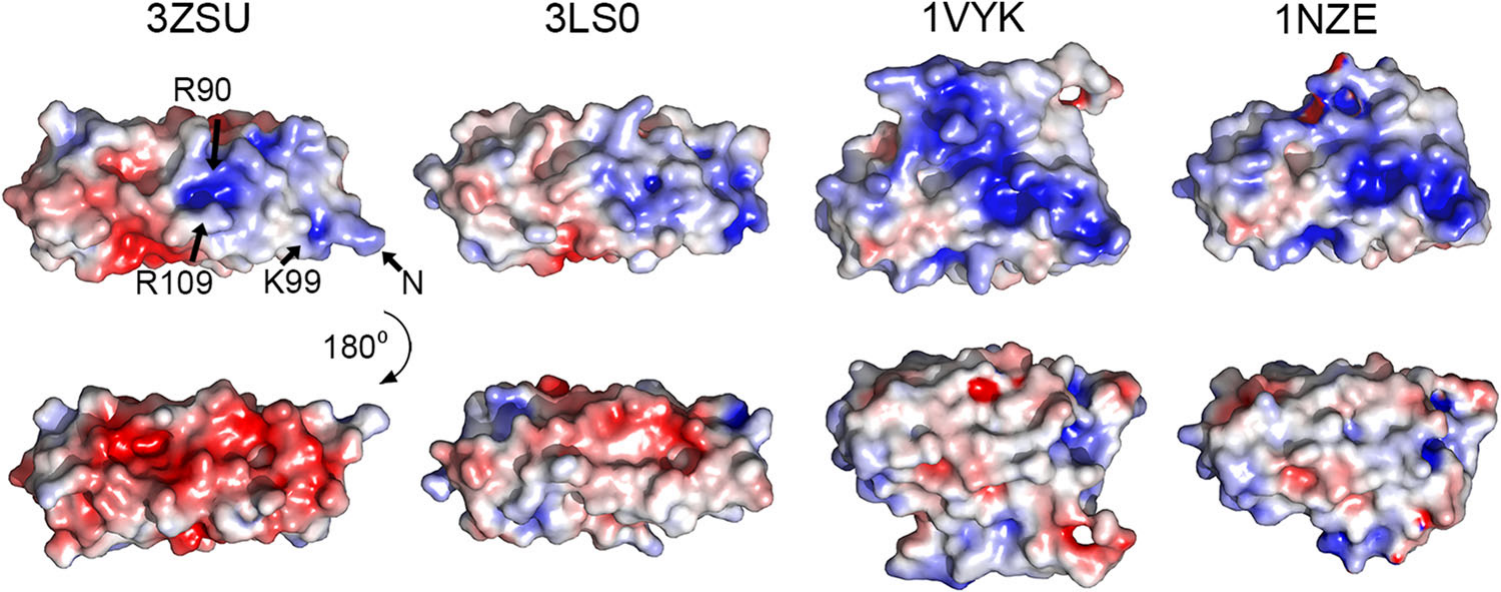
Solvent accessible surface charges of CyanoQ from *T. elongatus* (3ZSU), *Synechocystis* (3LS0) and spinach PsbQ (1VYK and 1NZE). Colour range spans from -5 (red) to 5 (blue) kT/e. Differences between the two spinach structures result from the fact that fewer residues could be fitted in 1NZE. Arrows point at C_α_ of selected residues. Arg_109_ is resolved in dual conformation

All three crystallised proteins differ in their isoelectric points as calculated by Protparam (Gasteiger et al. 2005) with pI values of 4.5 for *T. elongatus* CyanoQ, 5.6 for *Synechocystis* CyanoQ and 9.25 for spinach PsbQ. This is reflected in their surface charge distribution (Fig. 5). Both CyanoQ proteins show only a small patch of positively charged surface around *T. elongatus* Arg_109_, whereas the equivalent region of the PsbQ protein contains a large patch of lysine residues thought to be involved in binding to PSII (Meades et al. 2005) (Fig. 5, top).

Significant differences in surface charge are also observed on the opposite faces of PsbQ and CyanoQ (Fig. 5): PsbQ is relatively uncharged whereas CyanoQ is negatively charged (Fig. 5, bottom row). Given the differences in composition of the extrinsic PSII subunits in cyanobacteria and plants, this face of the protein may be involved in interactions with these subunits or with assembly factors or possibly other protein components in the thylakoid membrane.

### Comparison of zinc-binding sites

Zinc ions have been shown to bind to plant PsbQ (Calderone et al. 2003; Balsera et al. 2005) and CyanoQ from *Synechocystis* (Jackson et al. 2010), although the binding sites are not conserved (Fig. S7). Zinc has also been shown to bind to plant PsbP (Kopecky et al. 2012) and CyanoP from *T. elongatus* (Michoux et al. 2010) and *Synechocystis* (Jackson et al. 2012). The physiological relevance of these metal binding sites is currently unknown. In *Synechocystis* CyanoQ two zinc ions are coordinated by six amino-acid residues (Fig. 3 and Fig. S7). Despite the fact that five out of the six corresponding positions are occupied by potential metal ligands in *T. elongatus* CyanoQ, no zinc cations are present in the crystal structure. Unlike *Synechocystis* CyanoQ, where it was possible to obtain both zinc-bound and metal-free structures, our attempts to crystallise *T. elongatus* CyanoQ with zinc failed. Although there were no bound Zn^2+^ ions in our structure, we were able to fit a sulphate ion into the electron density. This anion is coordinated by three consecutive residues, Ser_126_ValThr_128_, found at the beginning of helix 4, at the apex of the protein.

### Possible binding sites for CyanoQ in PSII

Very recent chemical cross-linking experiments have suggested that *Synechocystis* CyanoQ might interact with both PsbO and CP47, at the interface of the two monomeric PSII complexes (Liu et al. 2014). The cross-linking data indicate that Asp_440_ of CP47 (numbering according to Liu et al. 2014) is in van der Waal’s contact with Lys_102_ of *Synechocystis* CyanoQ, and that Lys_120_ of *Synechocystis* CyanoQ is within 12 Å of both Lys_59_ and Lys_180_ of PsbO. Although Asp_440_ of CP47 is conserved in both *Synechocystis* and *T. elongatus*, Lys_102_ and Lys_120_ of *Synechocystis* CyanoQ are replaced by Thr_105_ and Asp_123_, respectively, in *T. elongatus* CyanoQ (3ZSU numbering) (Fig. S8). These cross-linked residues in CyanoQ are found in a region containing helices α2a, α2b and α3 and the H2-H3 cavity (Jackson et al. 2010) (Fig. 4). Highly conserved residues Arg_79_ and Asp_119_ found in the H2–H3 cavity highlighted in Fig. 4d are therefore good candidates for interacting with PsbO, whereas residue Gln_101_ might interact with CP47 (Fig. S8).

In contrast, a recent structural analysis of the isolated PSII complex from the red alga *Cyanidioschyzon merolae* suggests that PsbQ’ binds near to CP43 (Krupnik et al. 2013) rather than CP47. Given the significant structural differences between PsbQ and CyanoQ with regard the N-terminus and surface charge, we do not yet exclude the possibility that PsbQ and CyanoQ bind at different locations in PSII.

## Summary

We have provided evidence that CyanoQ binds to PSII complexes isolated from the thermophilic cyanobacterium *T. elongatus*, although the degree of association is dependent on the purification method. The crystal structures of CyanoQ and spinach PsbQ are very similar despite limited sequence identity with a four-helix bundle the common structural feature. This robust fold is likely to be conserved in the other members of the PsbQ family. Changes in the surface properties through mutation would explain how binding specificity could be altered to allow PsbQ-like proteins to bind outside PSII.

## Electronic supplementary material

Below is the link to the electronic supplementary material.

Supplementary material 1 (TIFF 506 kb)

Supplementary material 2 (TIFF 2756 kb)

Supplementary material 3 (TIFF 284 kb)

Supplementary material 4 (TIFF 2750 kb)

Supplementary material 5 (TIFF 391 kb)

Supplementary material 6 (TIFF 672 kb)

Supplementary material 7 (TIFF 266 kb)

Supplementary material 8 (TIFF 1616 kb)

Supplementary material 9 (TIFF 2041 kb)
