# Supplementary figures and images for: Crystal structure of CyanoQ from the thermophilic cyanobacterium Thermosynechococcus elongatus and detection in isolated photosystem II complexes
Source: Photosynth Res. 2014 May 18;122(1):57–67. doi: 10.1007/s11120-014-0010-z (PMC4180030; doi:10.1007/s11120-014-0010-z)

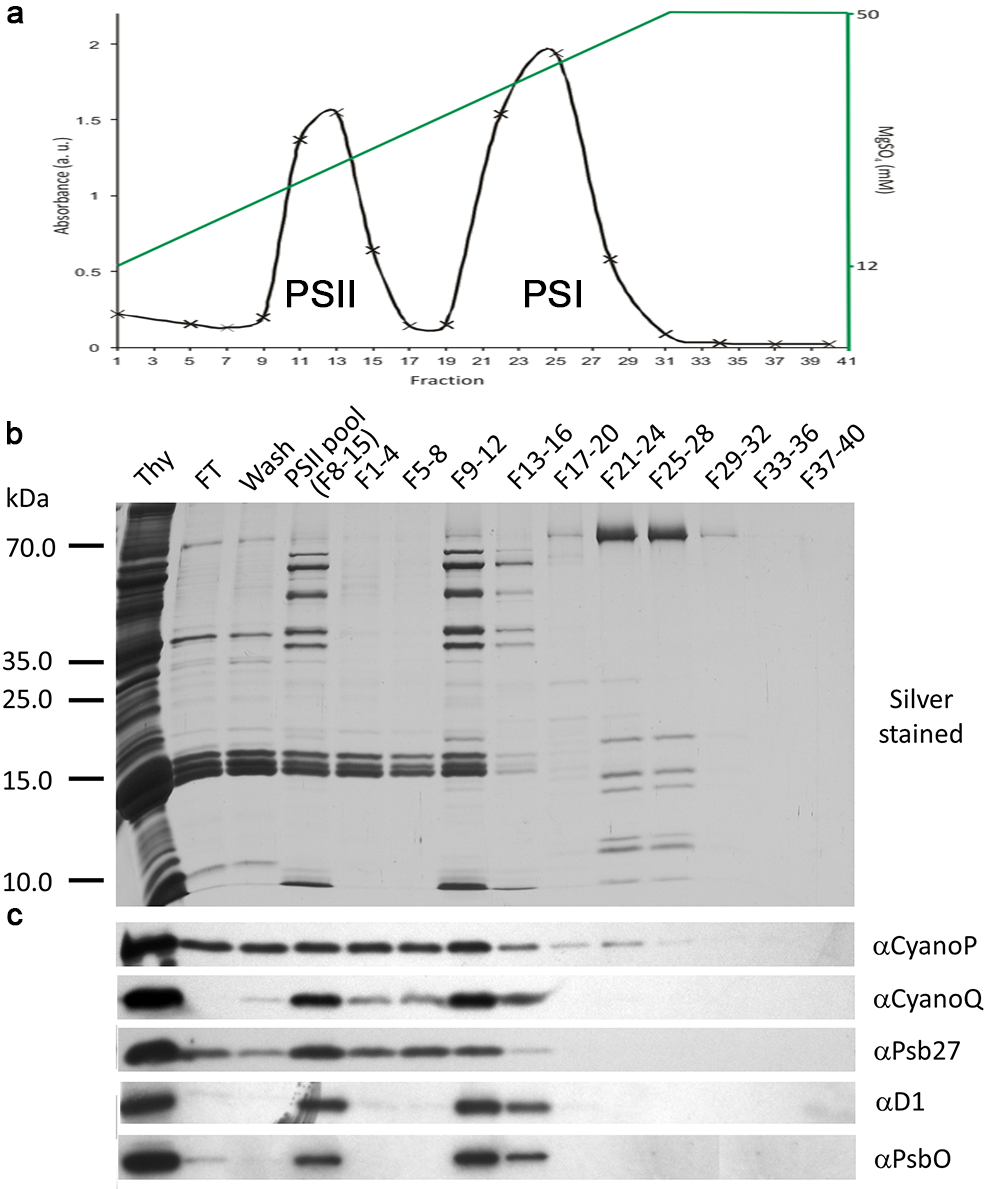

Supplement: Supplementary file 1 — Supplementary material 1 (TIFF 506 kb) [file 11120_2014_10_MOESM1_ESM.tif]

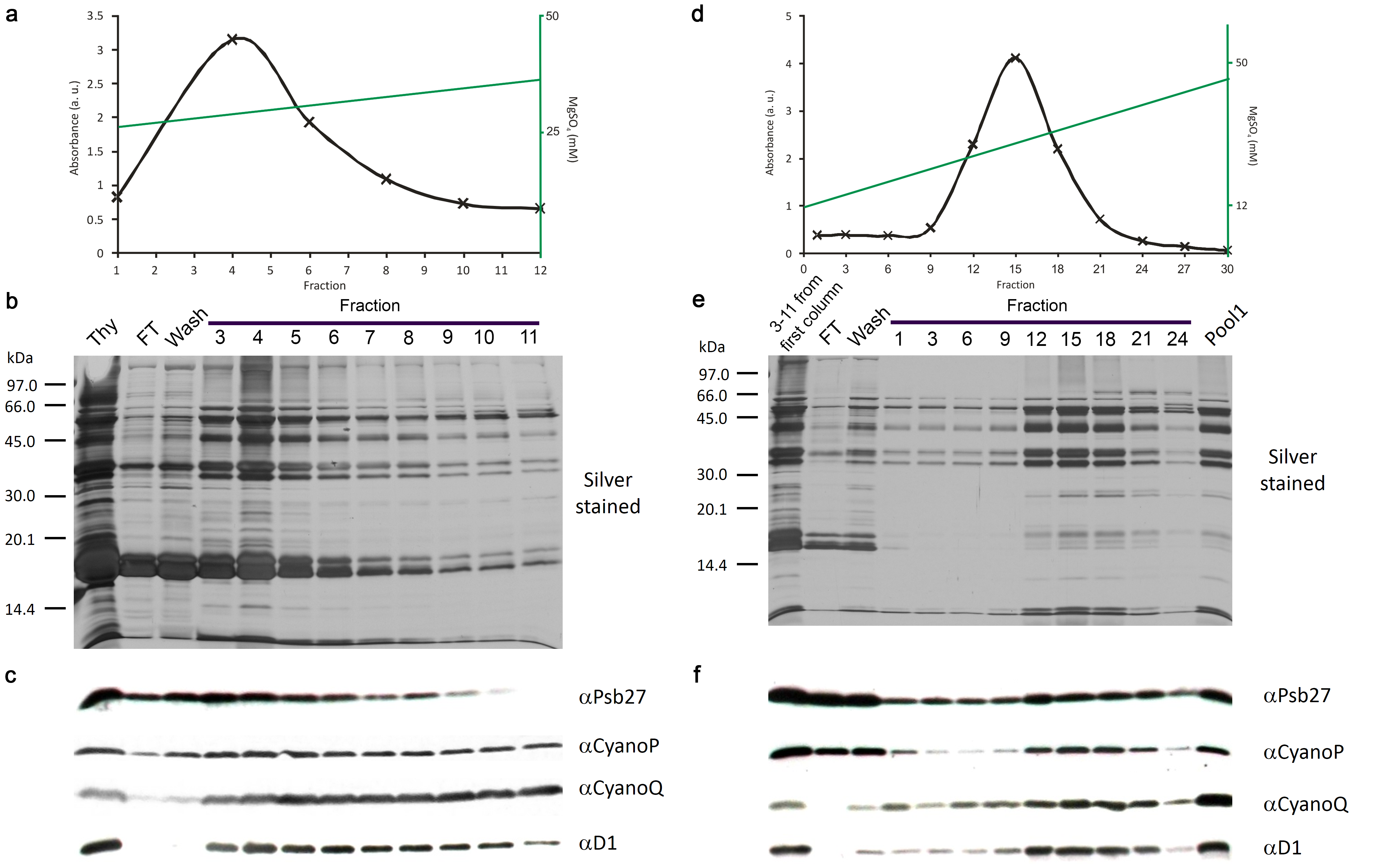

Supplement: Supplementary file 2 — Supplementary material 2 (TIFF 2756 kb) [file 11120_2014_10_MOESM2_ESM.tif]

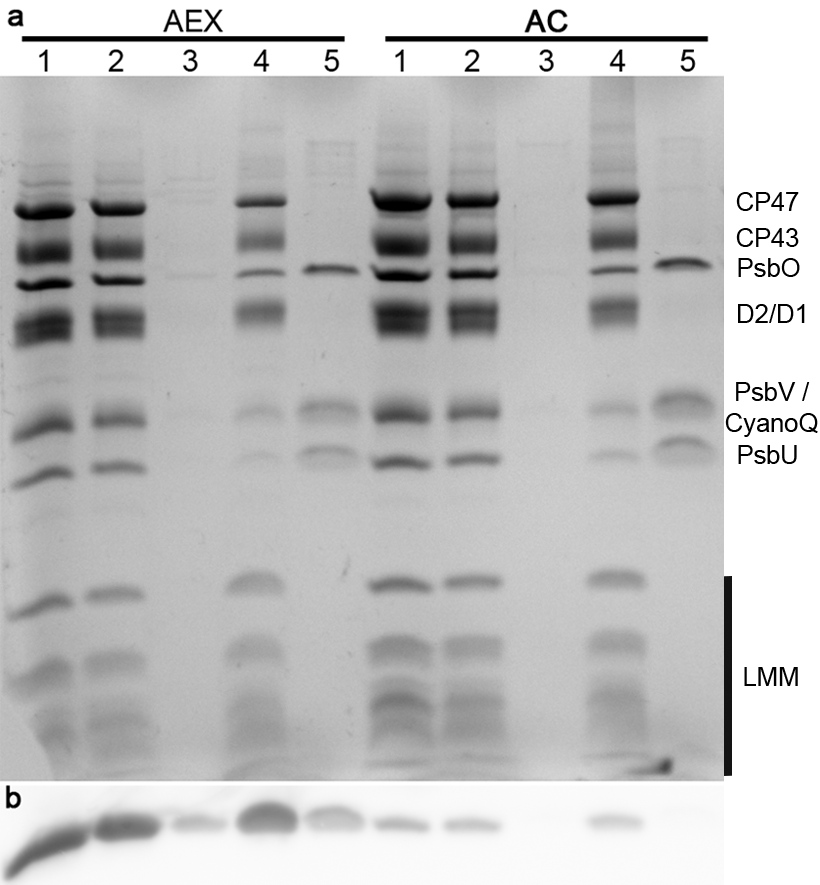

Supplement: Supplementary file 3 — Supplementary material 3 (TIFF 284 kb) [file 11120_2014_10_MOESM3_ESM.tif]

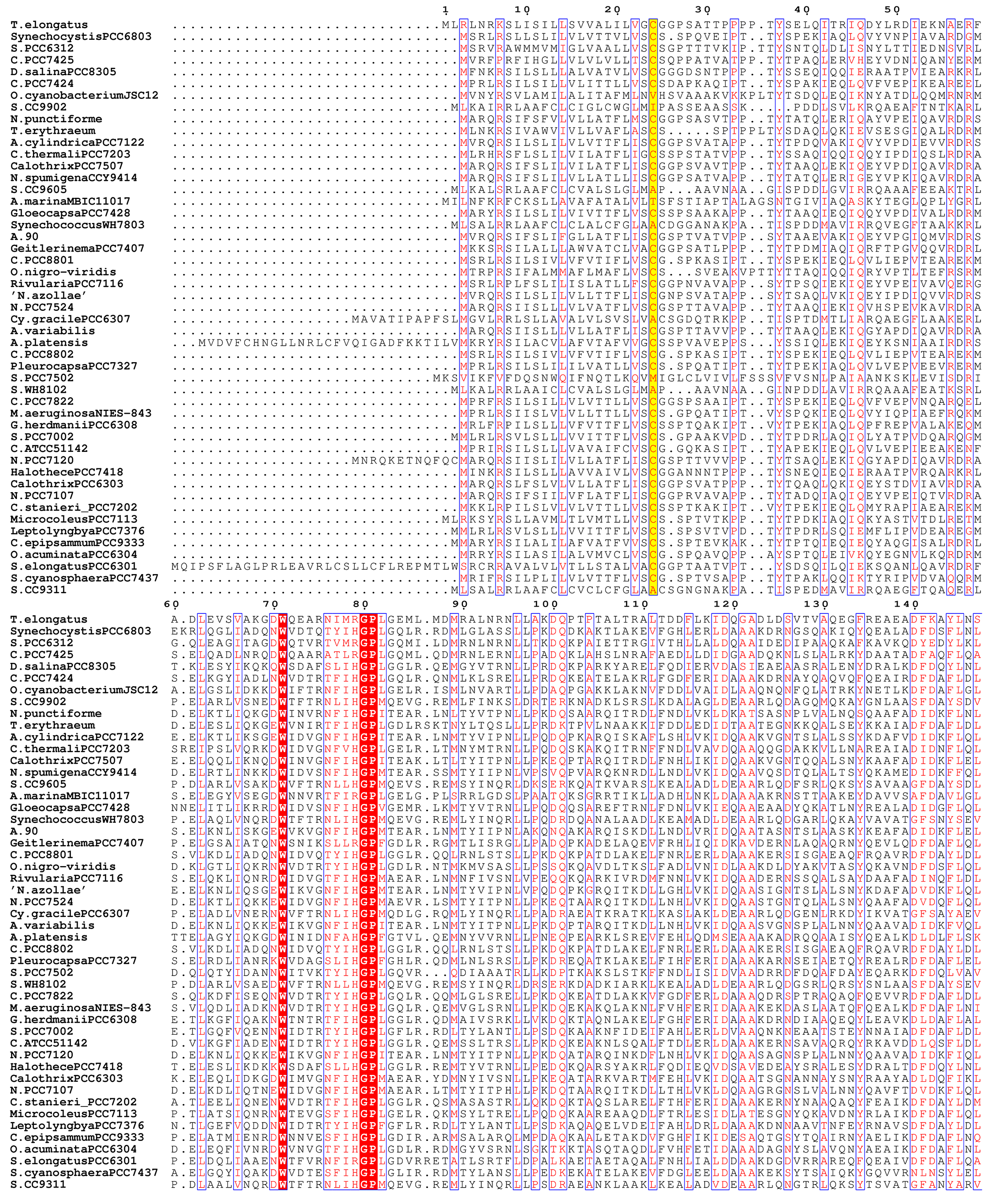

Supplement: Supplementary file 4 — Supplementary material 4 (TIFF 2750 kb) [file 11120_2014_10_MOESM4_ESM.tif]

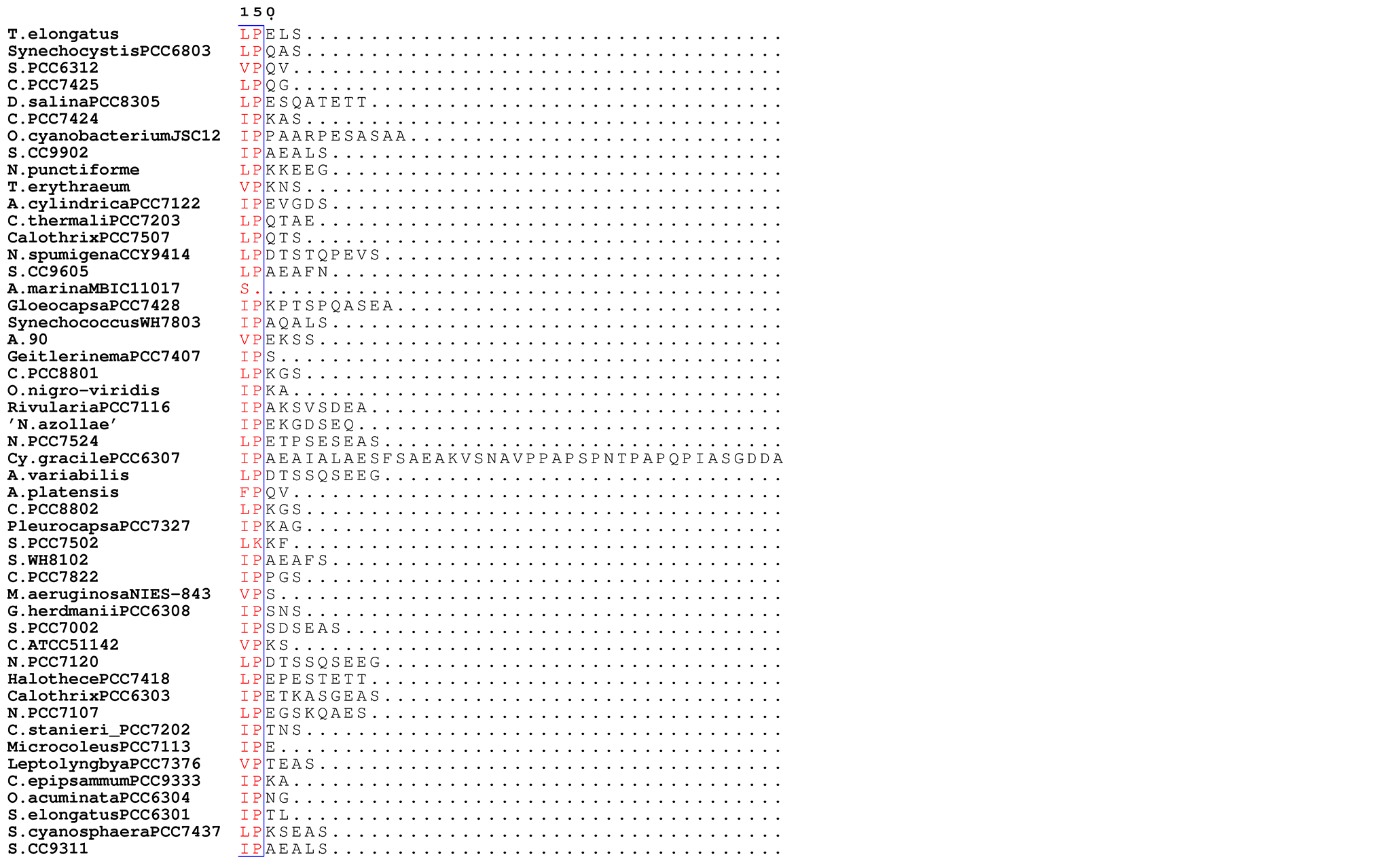

Supplement: Supplementary file 5 — Supplementary material 5 (TIFF 391 kb) [file 11120_2014_10_MOESM5_ESM.tif]

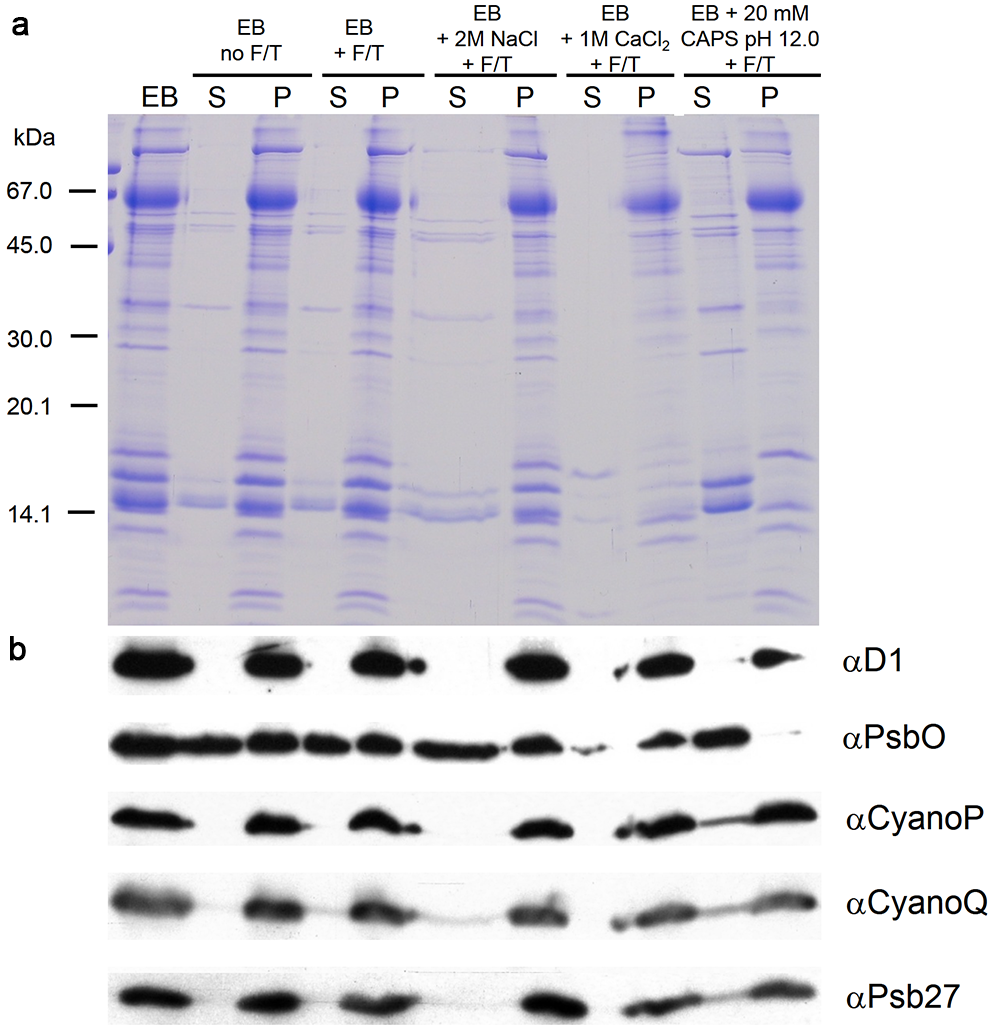

Supplement: Supplementary file 6 — Supplementary material 6 (TIFF 672 kb) [file 11120_2014_10_MOESM6_ESM.tif]

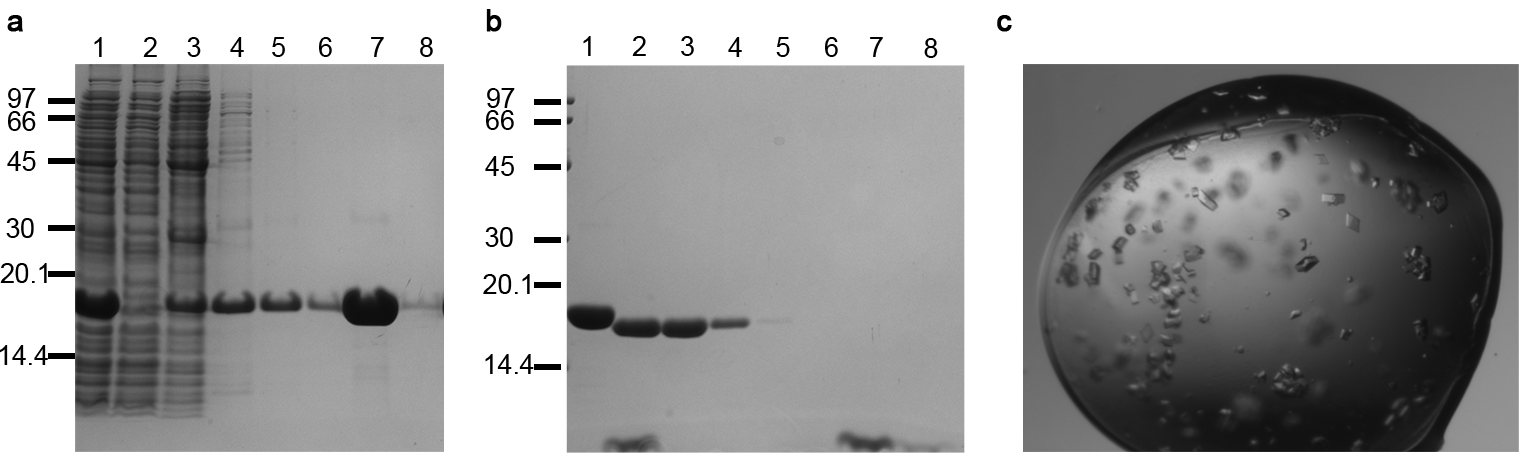

Supplement: Supplementary file 7 — Supplementary material 7 (TIFF 266 kb) [file 11120_2014_10_MOESM7_ESM.tif]

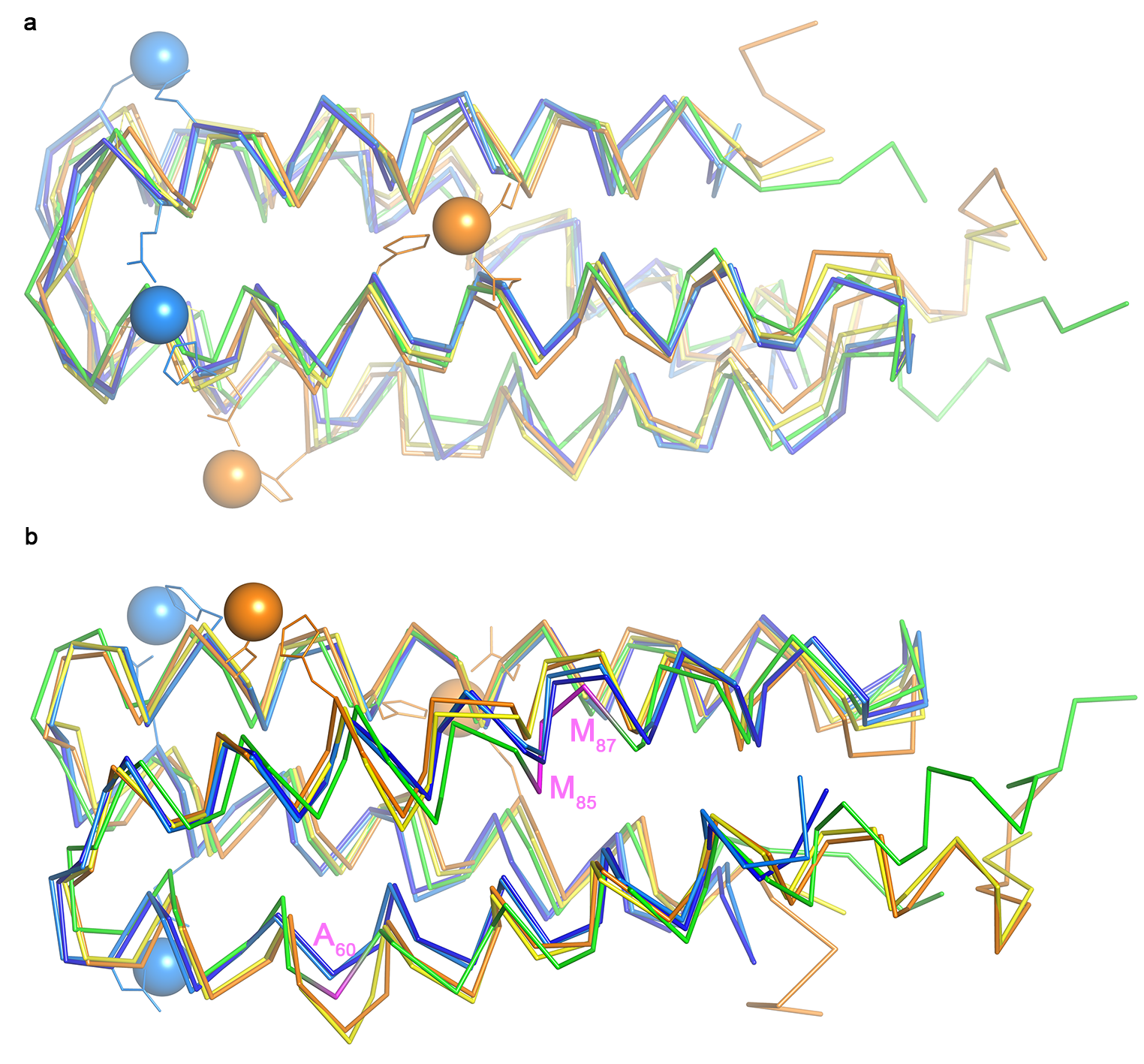

Supplement: Supplementary file 8 — Supplementary material 8 (TIFF 1616 kb) [file 11120_2014_10_MOESM8_ESM.tif]

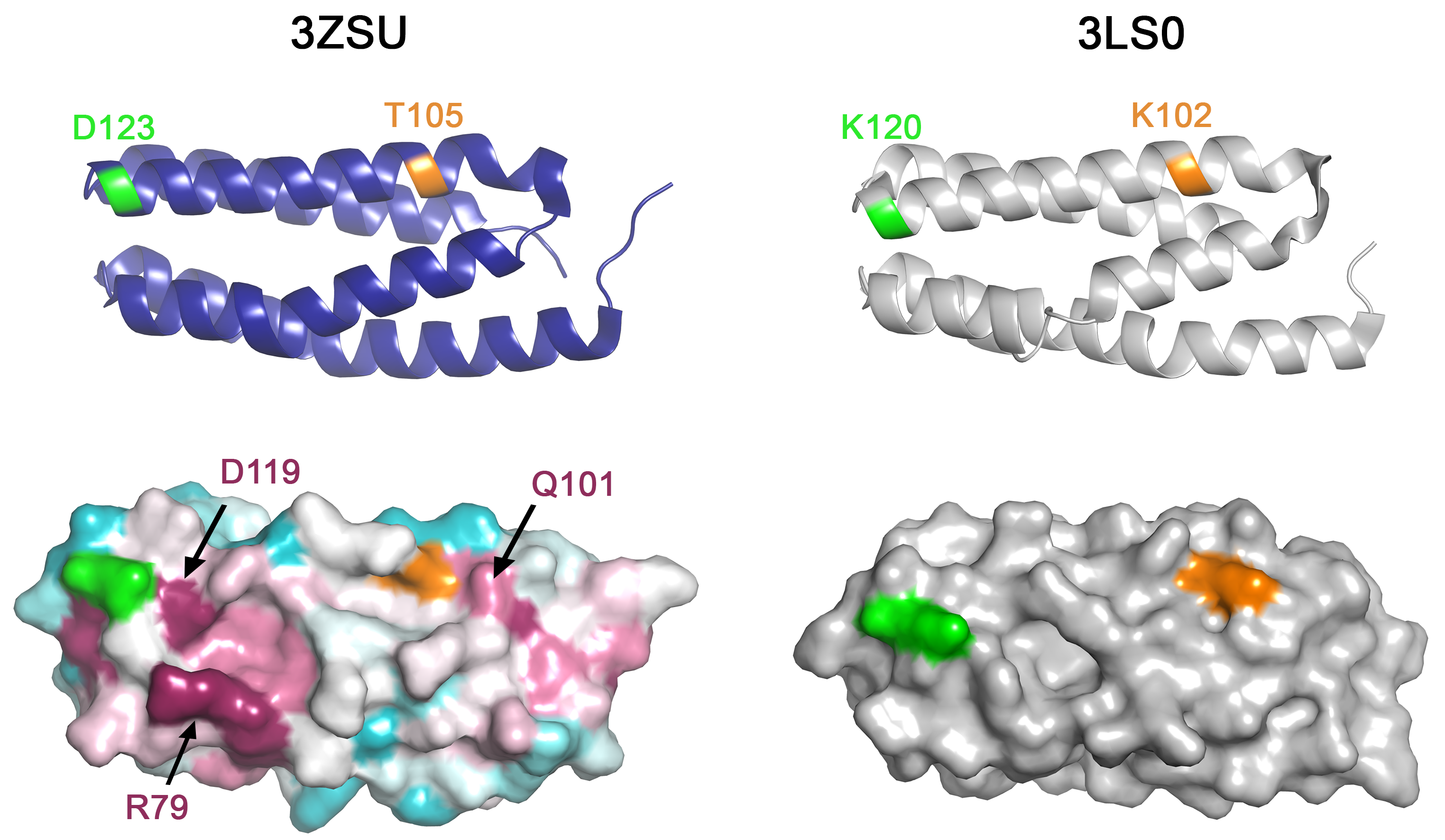

Supplement: Supplementary file 9 — Supplementary material 9 (TIFF 2041 kb) [file 11120_2014_10_MOESM9_ESM.tif]
